# Research to Accelerate Statewide Movements on Age-Friendly Communities

**DOI:** 10.1093/geroni/igaf122.1108

**Published:** 2025-12-31

**Authors:** Emily Greenfield, Megan Wolfe

## Abstract

This symposium will showcase the use of various research methods, mobilized through public-private partnership, to accelerate statewide movements on age-friendly communities. The first presentation will highlight the University of California, Davis, Family Caregiving Institute’s role as technical assistance providers, evaluators, and thought partners for the California Department of Aging’s (CDA) statewide Local Aging and Disability Action Planning (LADAP) grant program. They will describe how both evaluation processes and findings amplified the program, with implications for cross-sectoral partnerships for community aging initiatives nationwide. The second presentation will feature the work of the Center for Social and Demographic Research on Aging at the University of Massachusetts, Boston. They will present how their findings from an environmental scan of age-friendly state work informed the “refresh” of the State Plan on Aging for Massachusetts. The third presentation will provide an overview of university-community partnerships in New Jersey with researchers at the Rutgers School of Social Work Hub for Aging Collaboration. This presentation will demonstrate how developmental evaluation methods applied to a private grantmaking program helped to catalyze the development of a state-supported age-friendly communities program. The final presentation will feature how the University of Minnesota’s Center for Healthy Aging and Innovation engaged communities to inform the multisector blueprint on aging as part of the Governor’s Council for an Age-Friendly Minnesota. The discussant will address how community-engaged research with partners at the state and local levels can expand network capacity for improving aging societies and accelerate age-friendly social change for both scientific and social impact.

